# Supplementary material for: Chromatix: a differentiable, GPU-accelerated wave-optics library
Source: bioRxiv. 2026 Mar 25:2025.04.29.651152. Preprint. [Version 2] doi: 10.1101/2025.04.29.651152 (PMC13042145; doi:10.1101/2025.04.29.651152)
Supplement: Supplement 1 — Fig. 1 | Chromatix enables parallelization of optical simulations with minimal code changes. For all methods, we show the optical model followed by its implementation using Chromatix in both an unparallelized single GPU simulation as well as the parallelized versions that run across multiple GPUs. This display demonstrates how Chromatix allows a researcher to describe an optical model independently of the code required to scale that model across multiple GPUs in parallel. Colors in the code denote the corresponding elements in the model diagrams on the left. Note that the code shown is not the complete code that is actually used in demonstrations across the rest of the paper; this code is meant to provide a high level depiction of Chromatix code and parallelization. Ellipses indicate code that has been intentionally omitted for brevity. Simulation results are identical for all of the parallelization scenarios demonstrated here. a, Optical model for quantitative phase imaging showing how a single simulation can be easily run independently on multiple GPUs. b, Optical model for 3D holography showing how a simulation can be run multiple times in parallel on one GPU, with this whole batch of simulations run in parallel across multiple GPUs. c, Optical model for 3D snapshot microscopy (the Holoscope) showing how a simulation can be run across one large 3D sample split across multiple GPUs to enable larger simulations/optimizations. [file media-1.pdf]

a 3D quantitative phase imaging

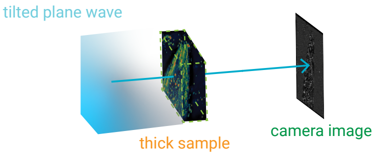


```
def quantitative_3d_phase(kykx):
    field = plane_wave(...)
    field = multislice_thick_sample(
        field, kykx=kykx, ...
    )
    image = basic_sensor(field, ...)
    return image
```

parallelize one image per GPU

```
@jax.pmap
def quantitative_3d_phase(kykx):
    field = plane_wave(...)
    field = multislice_thick_sample(
        field, kykx=kykx, ...
    )
    image = basic_sensor(field, ...)
    return image
```

b 3D holography in free space

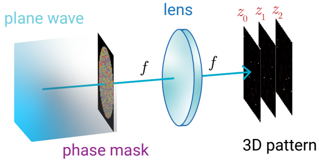


```
def holography_3d(phase_mask):
    field = plane_wave(...)
    field = phase_change(field  phase_mask)
    field = ff_lens(field, ...)
    field = transfer_propagate(field, z)
    return field
```

parallelize multiple targets per GPU

```
@jax.pmap
@jax.vmap
def holography_3d(phase_mask):
    field = plane_wave(...)
    field = phase_change(field, phase_mask)
    field = ff_lens(field, ...)
    field = transfer_propagate(field, z)
    return field
```

c 3D snapshot microscopy (Holoscope)

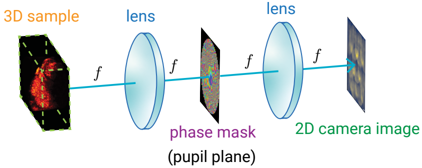


```
def holoscope_psf(phase_mask):
    field = objective_point_source(...)
    field = phase_change(field, phase_mask)
    field = ff_lens(field, ...)
    return field.intensity

microscope = Microscope(
    holoscope_psf(..., phase_mask),
    BasicSensor(...,),
    ...,
)

def holoscope(sample):
    image = microscope(sample)
    return image
```

split one volume across GPUs

```
def holoscope_psf(phase_mask):
    field = objective_point_source(...)
    field = phase_change(field, phase_mask)
    field = ff_lens(field, ...)
    return field.intensity

microscope = Microscope(
    holoscope_psf(..., phase_mask),
    BasicSensor(
        ...,
        reduce_parallel_axis_name="devices"
    ),
    ...,
)

@partial(jax.pmap)
def holoscope(sample, phase_mask):
    image = microscope(sample)
    return image
```
