# Supplementary figures and images for: Chromatix: a differentiable, GPU-accelerated wave-optics library
Source: bioRxiv. 2026 Mar 25:2025.04.29.651152. Preprint. [Version 2] doi: 10.1101/2025.04.29.651152 (PMC13042145; doi:10.1101/2025.04.29.651152)

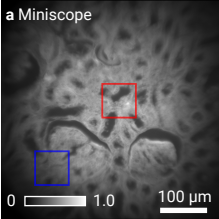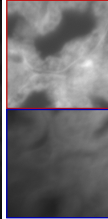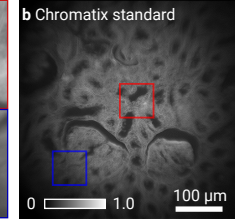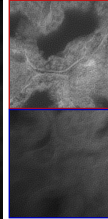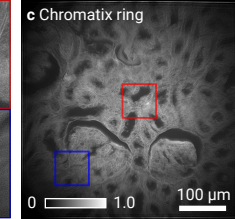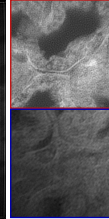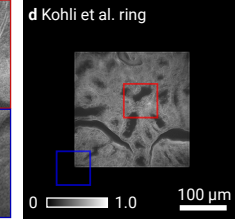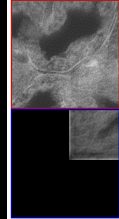

Supplement: Supplement 2 — Fig. 2 | Reconstruction performance of ring deconvolution without correction of vignetting. Highlighted regions in a - d show zoomed in cutouts from the center (red) and edge (blue) of the field of view on the bottom row. Intensity values in a - d are normalized. Measured image of incoherently illuminated rabbit liver a from a Miniscope with a GRIN lens showing extreme spatially-varying aberrations across the field of view; b, Chromatix rotationally invariant deconvolution demonstrating improved image quality across the entire field of view; c, Spatially invariant (standard) deconvolution demonstrating good quality in the center but degraded quality in the edges of the field of view; and d, original PyTorch rotationally invariant deconvolution which does not parallelize and cannot fit the whole field of view on 1 H100 GPU (80 GB memory). [file media-2.pdf]

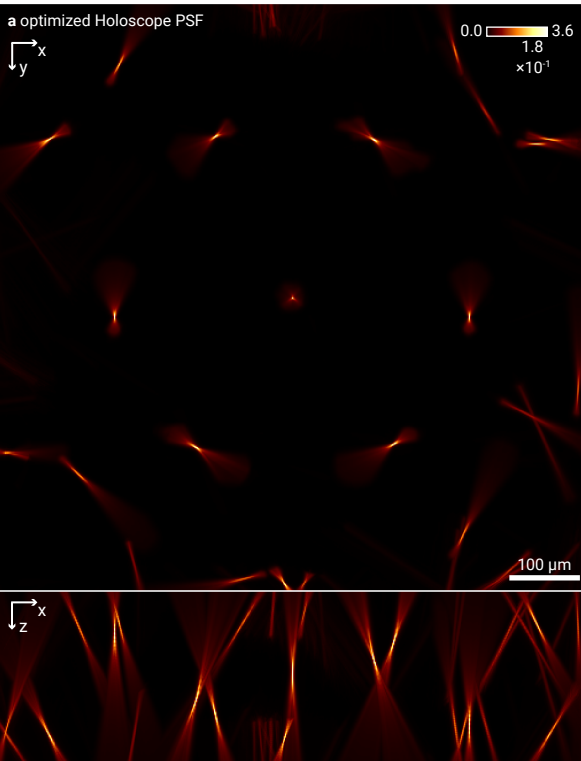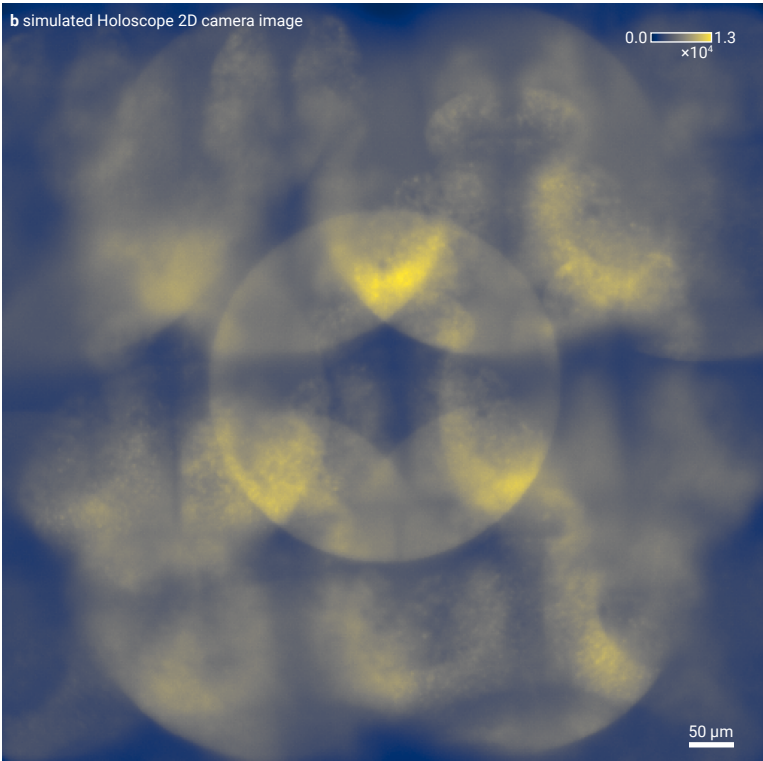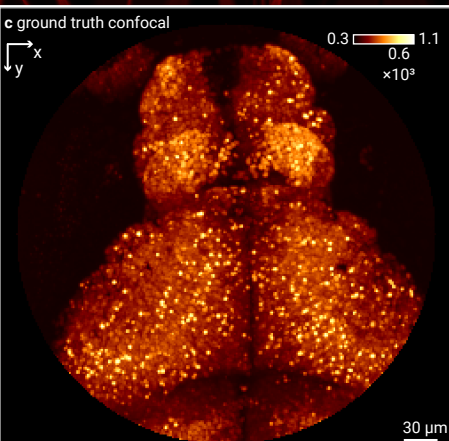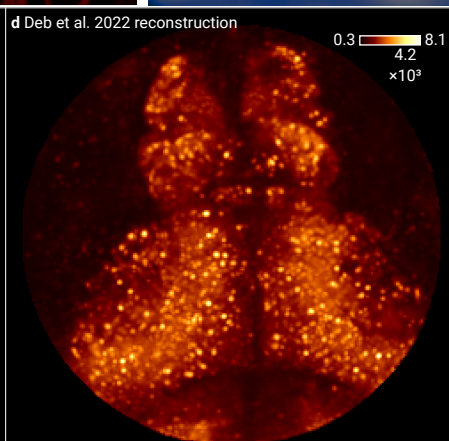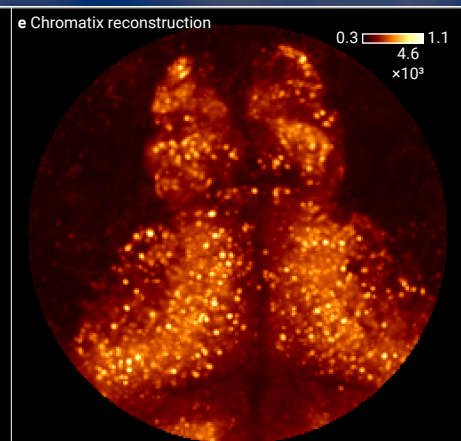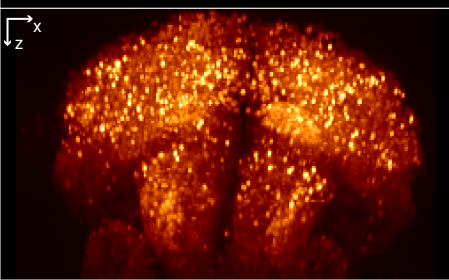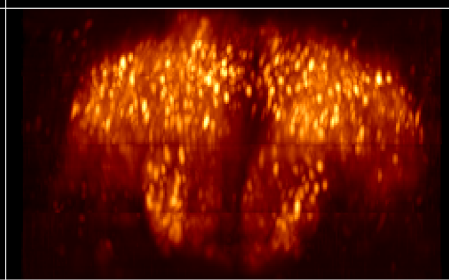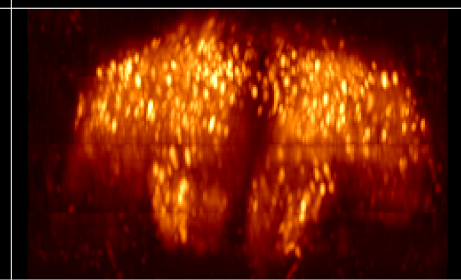

Supplement: Supplement 4 — Fig. 4 | Chromatix matches the in silico Holoscope reconstruction performance of Deb et al. a, Holoscope PSF optimized for zebrafish with a circular aperture in the original implementation by Deb et al.3 b, Simulated camera image showing a 3D zebrafish sample captured in a single 2D image. c, Ground truth confocal volume of the brain of a larval zebrafish (D. rerio), which will be over an order of magnitude slower to capture than the single snapshot image in (b). d, Reconstruction using the original implementation by Deb et al.3 e, Reconstruction using Chromatix, which matches the reconstruction performance of the original implementation despite being significantly faster computationally as shown in Figure 5. On a test set of 10 volumes, reconstruction networks trained with identical PSFs offer an SSIM of 0.979 ± 0.003 (not significantly different at p = 0.695 via two-sided t-test) for both Chromatix and the original implementation3 and a peak signal to noise ratio (PSNR) of 35.91 ± 0.92 for Chromatix versus 36.99 ± 1.07 for the original implementation3 (not significantly different at p = 0.063 via two-sided t-test). For both SSIM and PSNR, higher is better. [file media-4.pdf]

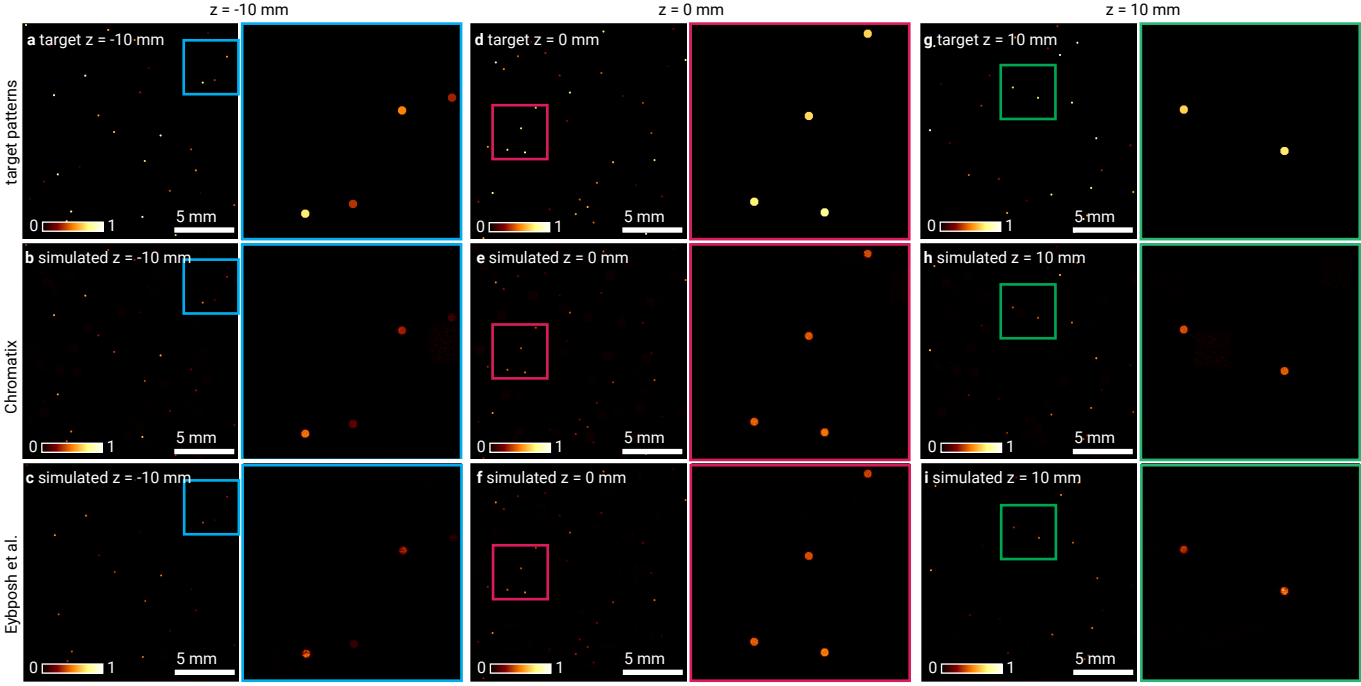

Supplement: Supplement 5 — Fig. 5 | Chromatix matches the in silico DeepCGH performance of Eybposh et al. a,d,g, Target pattern of dots of random intensity at planes -10 mm, 0 mm, and 10 mm from the focal plane respectively. b,e,h, Simulated pattern using holograms generated by a DeepCGH12 model trained using Chromatix at planes −10 mm, 0 mm, and 10 mm from the focal plane respectively. c,f,i, Simulated pattern using holograms generated by a DeepCGH model trained using the original implementation by Eybposh et al.12 at planes -10 mm, 0 mm, and 10 mm from the focal plane respectively. On a test set of 16 target patterns, we obtain an SSIM of 0.985 ± 0.001 for Chromatix versus 0.982 ± 0.001 for the original implementation12 (significantly different at p = 0.018 < 0.05 via two-sided t-test) and a PSNR of 35.40 ± 0.37 for Chromatix versus 34.95 ± 0.16 for the original implementation12 (not significantly different at p = 0.177 via two-sided t-test). For both SSIM and PSNR, higher is better. [file media-5.pdf]
