# Supplementary material for: Chromatix: a differentiable, GPU-accelerated wave-optics library
Source: bioRxiv. 2026 Mar 25:2025.04.29.651152. Preprint. [Version 2] doi: 10.1101/2025.04.29.651152 (PMC13042145; doi:10.1101/2025.04.29.651152)
Supplement: Supplement 3 — Fig. 3 | Chromatix outperforms the original CoCOA implementation for estimating aberrations on fluorescent bead data. a, Widefield measurement of fluorescent beads, showing aberrations and high background. b, Kang et al.47 reconstruction of the fluorescent beads. c, Chromatix reconstruction of the fluorescent beads. d, Ground truth aberrated wavefront based on the imposed Zernike modes. e, Kang et al.47 estimation of the aberrated wavefront. f, Chromatix estimation of the aberrated wavefront. g, Bar plot comparing the wavefront error (nm) between the true imposed coefficients and the estimated coefficients for each Zernike mode across both Chromatix (blue) and the original implementation47 (red). Note that all Zernike modes aside from 5, 6, and 7 should have a coefficient of 0 nm in the imposed aberration. h, Bar plot of root mean square (RMS) of the difference between the true wavefront coefficients and the estimated coefficients across the three non-zero Zernike coefficients of the imposed aberration, demonstrating that Chromatix has approximately half the error compared to the Kang et al. implementation47, i, Bar plot of RMS of the pixelwise difference between the estimated aberrated wavefront and the true imposed wavefront, showing the wavefront recovered by Chromatix is more accurate to the ground truth than the original implementation47. [file media-3.pdf]

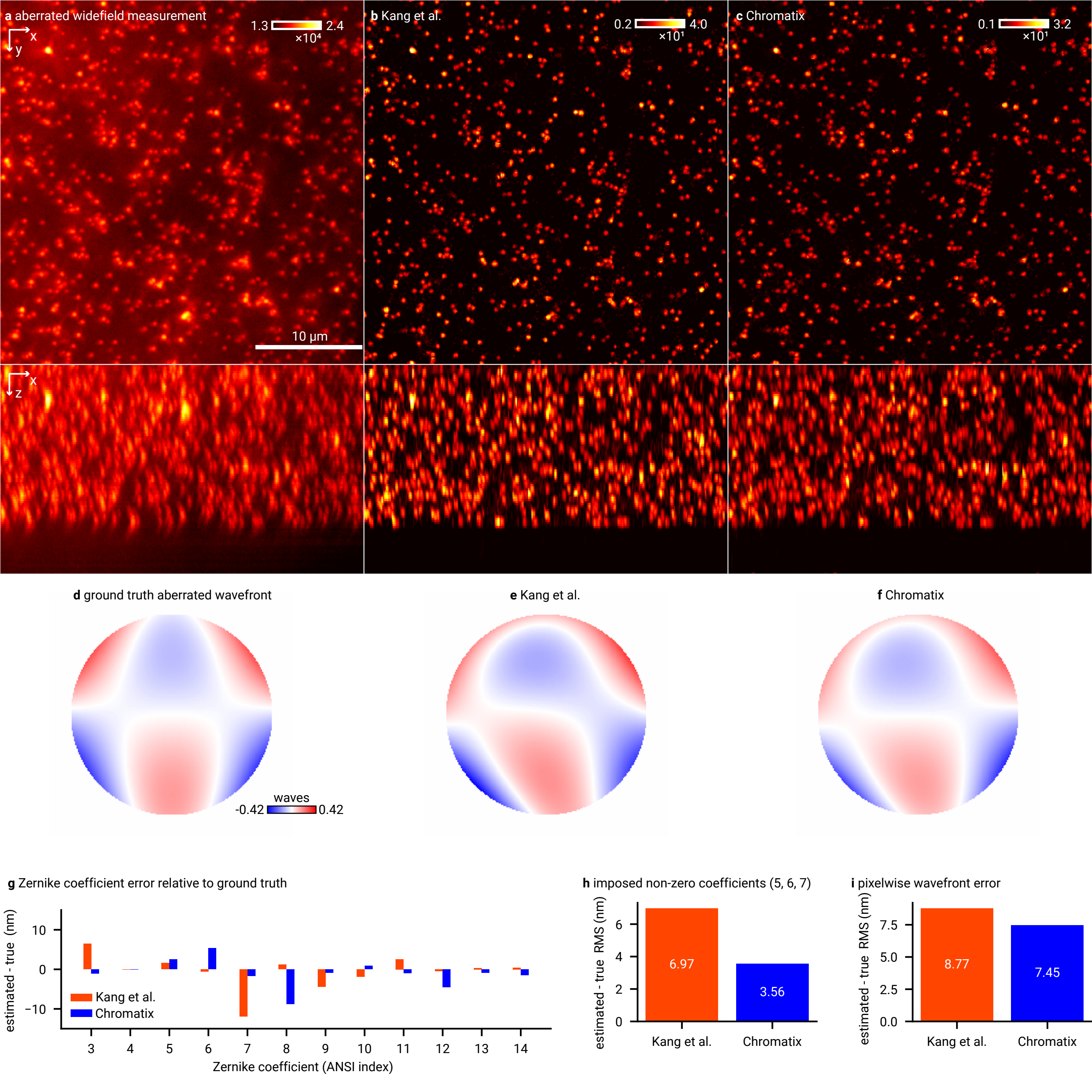
a aberrated widefield measurement
1.3 2.4 ×10⁴
x y
10 µm
x z
b Kang et al.
0.2 4.0 ×10¹
c Chromatix
0.1 3.2 ×10¹
d ground truth aberrated wavefront
waves
-0.42 0.42
e Kang et al.
f Chromatix
g Zernike coefficient error relative to ground truth
estimated - true (nm)
10
0
−10
Kang et al.
Chromatix
3 4 5 6 7 8 9 10 11 12 13 14
Zernike coefficient (ANSI index)
h imposed non-zero coefficients (5, 6, 7)
estimated - true RMS (nm)
0 2 4 6
6.97
3.56
Kang et al. Chromatix
i pixelwise wavefront error
estimated - true RMS (nm)
0.0 2.5 5.0 7.5
8.77
7.45
Kang et al. Chromatix
