# Supplementary material for: Chromatix: a differentiable, GPU-accelerated wave-optics library
Source: bioRxiv. 2026 Mar 25:2025.04.29.651152. Preprint. [Version 2] doi: 10.1101/2025.04.29.651152 (PMC13042145; doi:10.1101/2025.04.29.651152)
Supplement: Supplement 6 [file media-6.pdf]

Table 1 | Comparison of features against other simulation libraries/software.

	**Chromatix**	dO[29]	Optiland[32]	dLux[31]	Zemax[27]	CODE V[28]	Lumerical FDTD[73]	XLumina[30]
complex / birefringent 3D samples	✓	☐	☐	☐	☐	☐	✓	☐
scalar wave propagation	✓	☐	☐*	✓	✓	✓	☐	✓
vectorial wave propagation	✓	☐	☐	☐	✓	✓	✓	✓
off-axis wave propagation	✓	☐	☐	✓	✓	✓	✓	☐
diffractive elements (DMDs, SLMs)	✓	☐	☐	✓	✓	✓	✓	✓
thick lenses/stacks of lenses	✓**	✓	✓	☐	✓	✓	☐	☐
real lens models/lens coatings	☐	☐	✓	☐	✓	✓	☐	☐
polarization	✓	☐	✓	☐	✓	✓	✓	✓
multi-GPU parallel	✓	☐	☐	✓	☐	☐	✓	✓
automatic differentiation	✓	✓	✓	✓	☐	☐	☐	✓
open-source	✓	✓	✓	✓	☐	☐	☐	✓
focus	microscopy, holography	lens design	optical design	astronomy	optical design	optical design	photonics	super resolution
primary simulation mode	wave optics	ray optics	ray optics	wave optics	ray optics	ray optics	time-domain wave optics	wave optics
framework / scripting language	JAX (Python)	PyTorch (Python)	PyTorch (Python)	JAX (Python)	Zemax Programming	Macro-PLUS	Lumerical Script	JAX (Python)

***** Optiland does not provide a range of general propagation methods but does include a method to compute point spread functions with diffraction effects via the Fourier transform[32].

****** Chromatix uses a paraxial approximation to model the effect of thick lenses/ray transfer matrices via the Collins integral[74,75], which will not capture the effects of large systems of thick lenses at the edges of a field as accurately as ray tracing[27–29] or beamlet propagation[27,28].
